# Uncommon Localization of Pyogenic Granuloma: A Case Report of Eyebrow Involvement

**DOI:** 10.7759/cureus.95098

**Published:** 2025-10-21

**Authors:** Abdulrahman Saleh Aldairi, Faris Alsaedi, Rana Al-Zaidi, Ibrahim Allihibi, Mohammad Hakim, Homaid Alotaibi

**Affiliations:** 1 Department of Dermatology, King Faisal Hospital, Ministry of Health, Makkah, SAU; 2 Department of Anatomic Pathology, King Faisal Hospital, Ministry of Health, Makkah, SAU

**Keywords:** benign vascular lesion, capillary hemangioma, eyebrow, lobular capillary hemangioma, pyogenic granuloma, shave excision

## Abstract

Pyogenic granuloma (PG) is a benign vascular tumor that most often arises on the gingiva, lips, and fingers, while eyebrow involvement is exceptionally uncommon. We describe a 50-year-old woman who developed a progressively enlarging, bleeding nodule on the right eyebrow that ultimately interfered with her vision. The lesion was managed with shave excision and electrocautery, and histopathological analysis confirmed the diagnosis of PG. The patient remained disease-free after one year of follow-up. This case underscores the importance of recognizing PG in rare anatomical locations and considering it in the differential diagnosis of vascular lesions of the eyebrow.

## Introduction

Pyogenic granuloma (PG), also referred to as lobular capillary hemangioma, is a benign vascular lesion that develops in the skin and mucosal surfaces [1]. Despite its historical terminology, the condition is neither infectious nor granulomatous in nature; histologically, it is composed of clusters of proliferating capillaries separated by fibrous septa [2,3]. Clinically, PG usually presents as a solitary, reddish, pedunculated growth that expands quickly and bleeds with minimal trauma [2,3]. It occurs most often in younger individuals, children, and pregnant women, commonly affecting the gingiva, lips, digits, and other facial sites [4-6]. Involvement of less typical sites is distinctly uncommon, and eyebrow localization has been described only sporadically in the literature [1,2]. These rare presentations may imitate other vascular or malignant lesions such as amelanotic melanoma, angiosarcoma, or bacillary angiomatosis, thereby complicating the diagnostic process [1]. Presenting unusual cases contributes to the clinical understanding and supports accurate recognition. Here, we report the case of a 50-year-old woman with a pyogenic granuloma arising from the eyebrow, an anatomical location rarely encountered in practice.

## Case presentation

**Figure 1:**
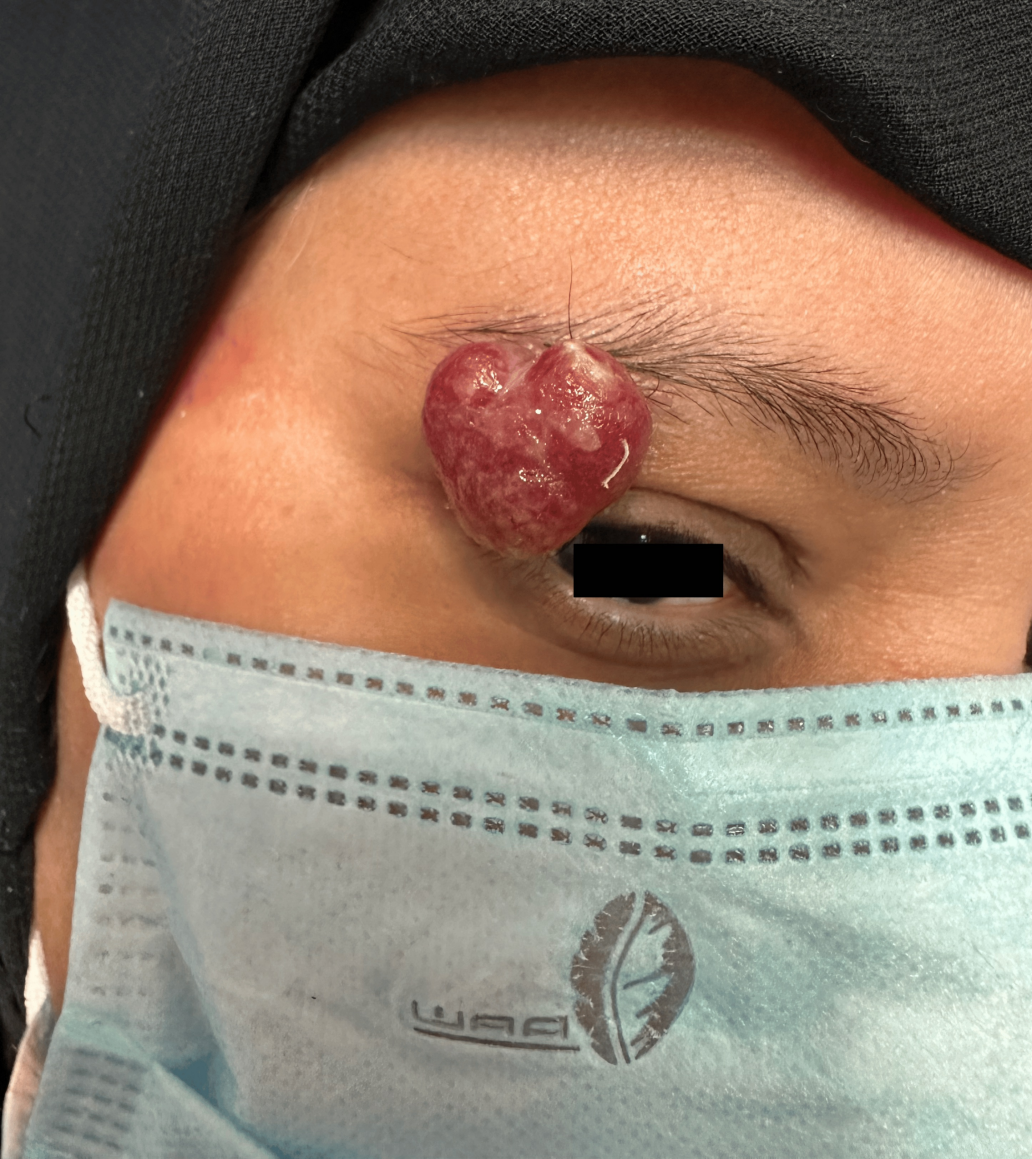
Pyogenic granuloma over the right eyebrow, obscuring the eyelid.

**Figure 2:**
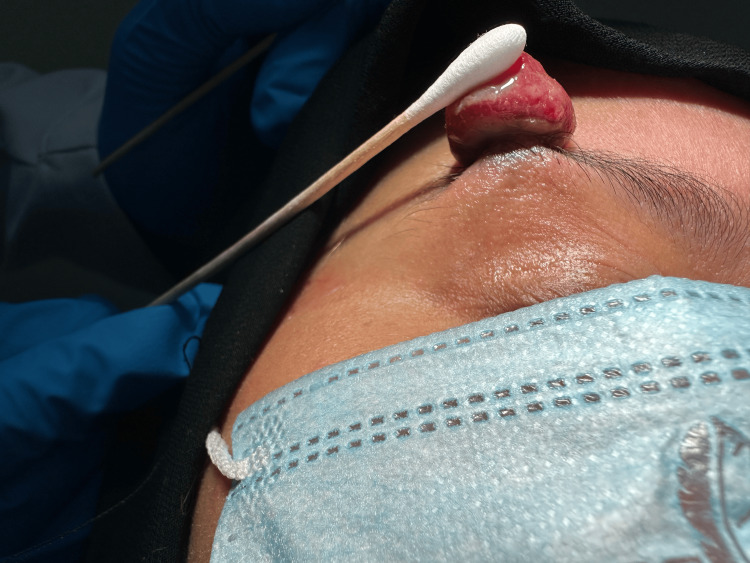
Pyogenic granuloma on the right eyebrow elevated with a cotton swab.

**Figure 3:**
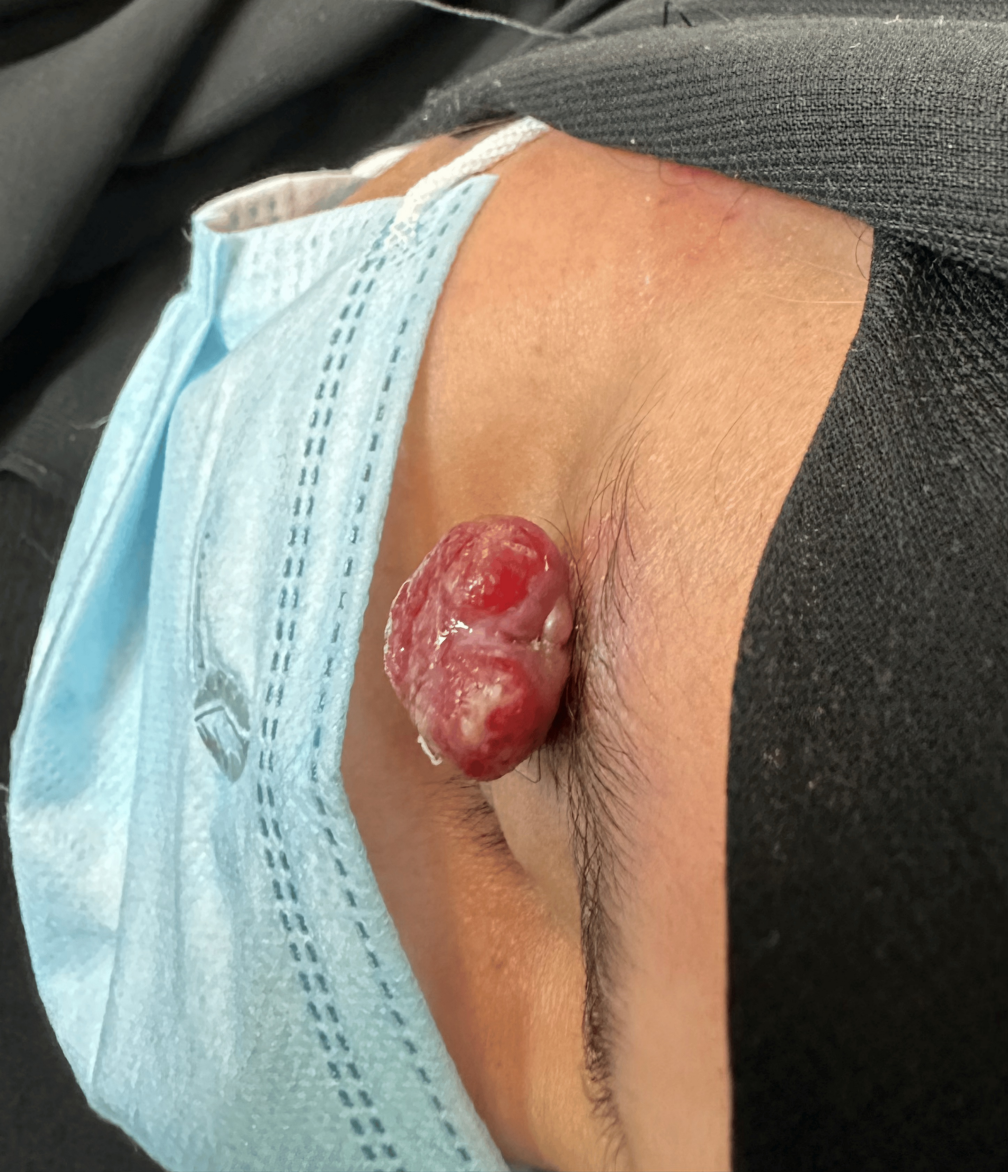
Pyogenic granuloma on the right eyebrow

A 50-year-old female patient with no prior medical conditions presented to the dermatology clinic with a painless, easily bleeding lesion over the right eyebrow that had persisted for 4 months. This was her first episode of such a lesion. She had previously attended several private clinics, where she was prescribed topical treatments, though she was unaware of their exact type and did not experience any benefit from them. Later, at a private clinic, she underwent an electrocautery procedure; however, the lesion subsequently recurred, enlarged, and caused repeated episodes of bleeding that required direct pressure to stop. In the few weeks leading up to her visit to our clinic, the lesion had shown progressive enlargement compared to earlier episodes, and it began to interfere with her daily activities by obscuring her vision due to its large size and downward extension over the right eye. A complete medical history was obtained and was unremarkable for trauma at the site or family history of similar lesions. The patient was not taking any medications. On the other hand, dermatological examination demonstrated a solitary, well-defined, exophytic, pedunculated, red to white, friable, lobulated nodule is seen arising from the right eyebrow region, measuring 2x1.5x1 cm. The surface appears smooth to slightly eroded and glistening (Figures 1-3).

**Figure 4:**
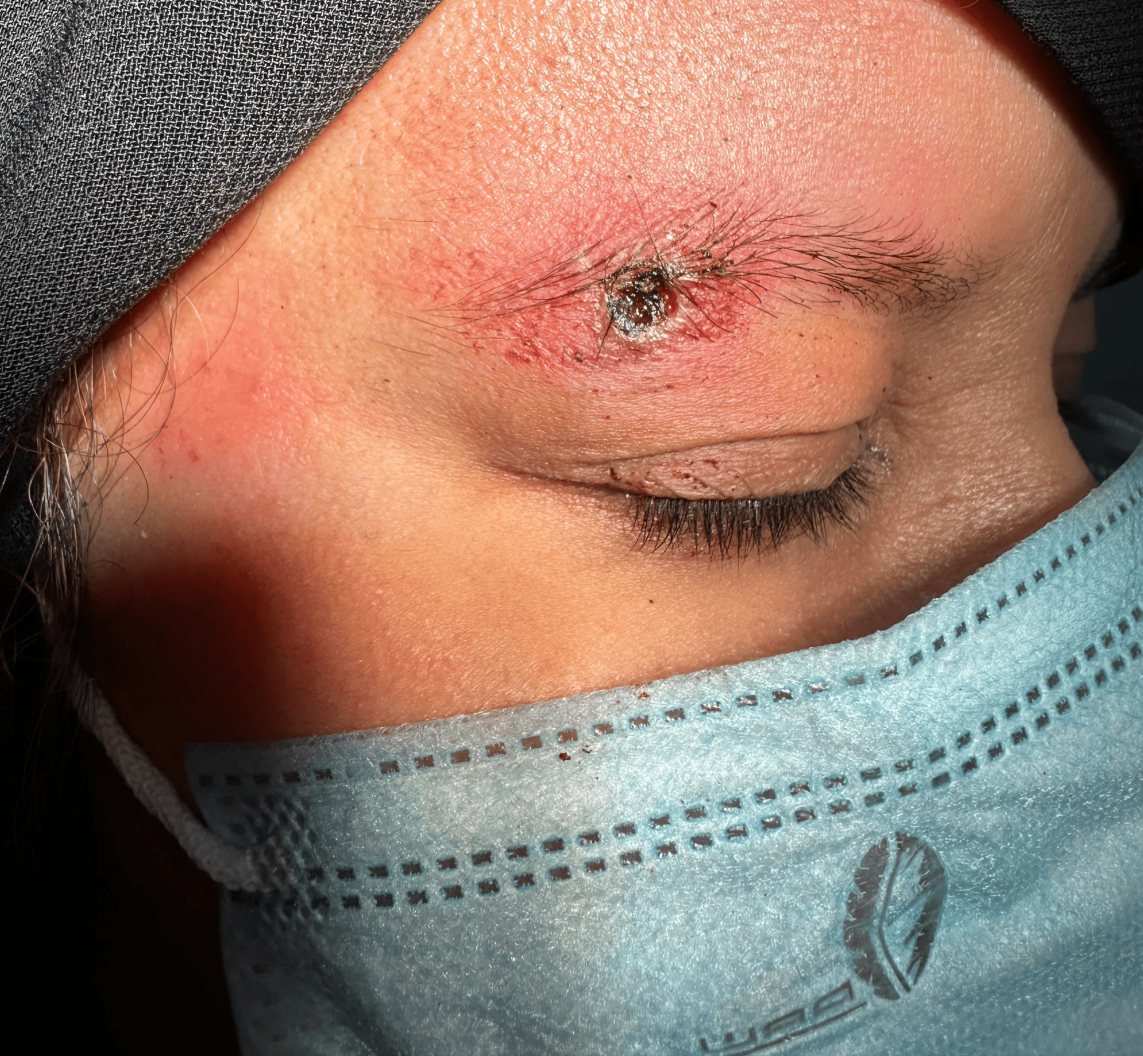
Post-shave excision site of the pyogenic granuloma on the right eyebrow.

A provisional diagnosis of pyogenic granuloma was made. Under aseptic technique, local anesthesia with 1% lidocaine was administered, followed by shave excision of the lesion using a flexible DermaBlade (Vedo Trade LLC, Las Vegas, USA). Electrocautery was also applied to minimize bleeding during the procedure (Figure 4).

**Figure 5:**
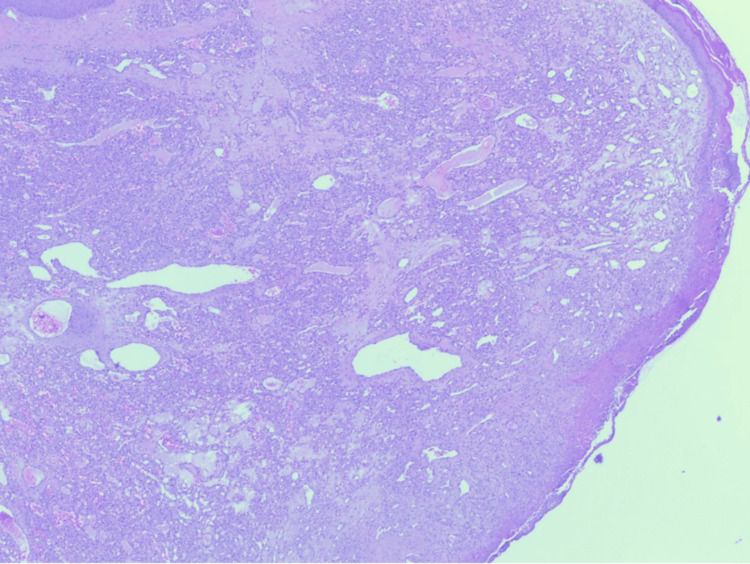
Low-power view of lobular capillary hemangioma showing a polypoid lesion comprised of lobules separated by fibrous septa. The lesion is covered by a focally ulcerated epidermis (hematoxylin-eosin stain, original magnifications x20).

**Figure 6:**
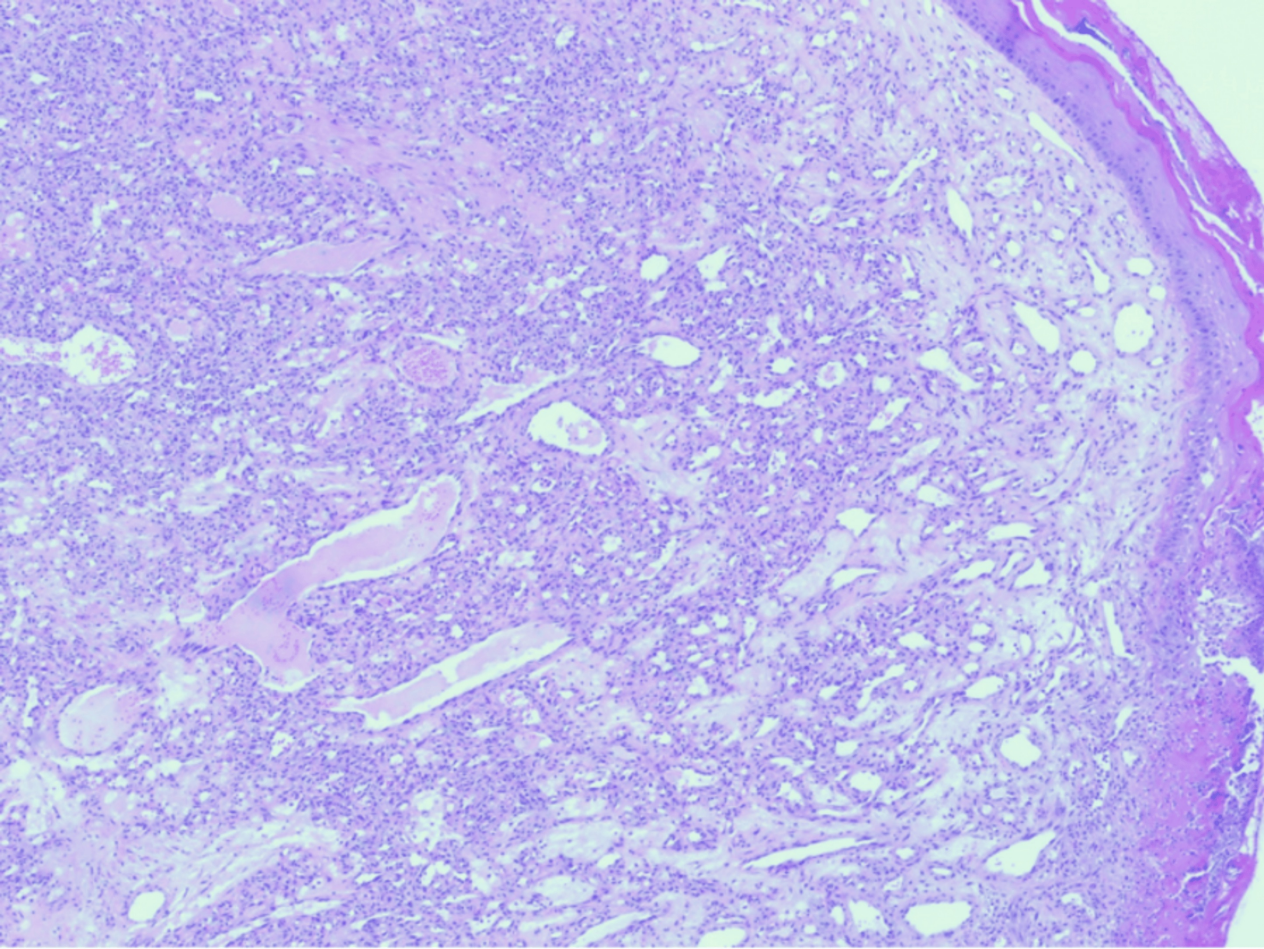
The lobules are formed of capillary-sized vascular channels with several large feeding vessels. The stroma is edematous with few mixed inflammatory cells (hematoxylin-eosin stain, original magnifications x40).

**Figure 7:**
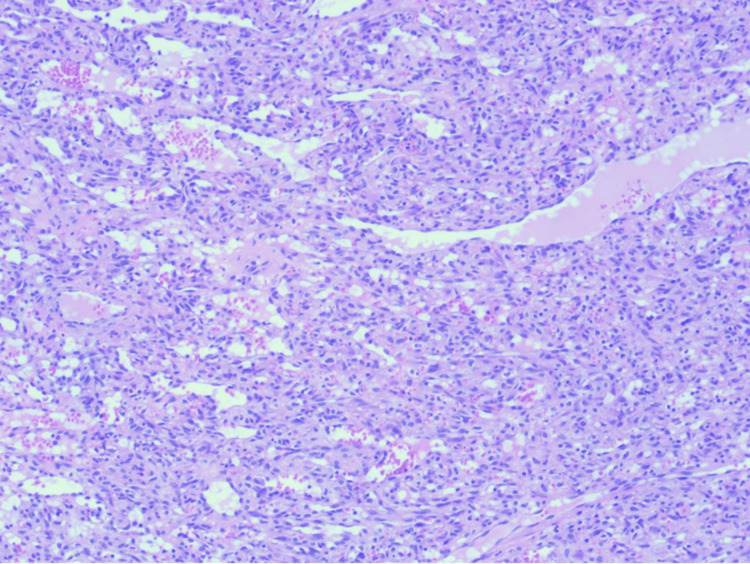
The vascular channels are lined by a single layer of bland endothelial cells, with rare typical mitotic figures (hematoxylin-eosin stain, original magnifications x100).

The excised tissue was submitted for histopathological evaluation. The histopathological findings revealed a polypoid lesion with surface ulceration covered by fibrinous exudate (Figure 5). The lesion shows small capillaries arranged in a lobular fashion (Figure 6). Endothelial cells of the lesion show no atypia (Figure 7), which confirmed the diagnosis of pyogenic granuloma. The patient was followed clinically for one year with no recurrence, after which she was discharged from the dermatology clinic.

## Discussion

Pyogenic granuloma is considered a reactive vascular tumor-like lesion with multifactorial origins, including minor trauma, hormonal fluctuations, drug exposure, and, in some cases, genetic alterations [2,6]. Certain pharmacologic agents such as retinoids (including isotretinoin) and BRAF inhibitors have been implicated in its development [5-8]. More recent molecular research has identified *BRAF *and *RAS *mutations in a subset of patients, reinforcing the concept of aberrant angiogenesis as a key mechanism [9]. This evolving evidence suggests that PG is not only reactive but may also involve molecular drivers of vascular proliferation.

Anatomically, PGs most often involve the gingiva, lips, and fingers, whereas eyebrow presentations are extremely uncommon [2]. In one retrospective review of 155 cases, only five lesions were located in the eyebrow region [2]. Likewise, Giblin et al. evaluated 408 cases and observed that most lesions occurred on oral and acral surfaces, while uncommon sites such as the eyebrow remained exceptional and required histological confirmation to exclude malignancies [7]. Similarly, Wollina et al. noted the rarity of periocular and eyebrow cases despite the otherwise broad anatomical distribution of PG [6]. A recurrent eyebrow PG with satellitosis has also been documented, further demonstrating the atypical potential of lesions in this region [1].

Histopathologically, PG is characterized by lobular capillary proliferation with endothelial cells lacking atypical changes [2,5]. This description was consistent with the findings in our case. Because PG can mimic malignant or infectious vascular disorders, biopsy is necessary for a definitive diagnosis [1,6].

Surgical excision remains the most effective treatment approach, with recurrence rates substantially lower compared with other methods [2,6,7]. Giblin et al. reported excellent results with excision across hundreds of cases [7]. Nevertheless, other treatment modalities - such as curettage, cryotherapy, electrocautery, sclerotherapy, and topical or systemic beta-blockers like timolol and propranolol - have also been explored, especially in cosmetically sensitive or pediatric cases [3,5,6,10]. Reviews, including that of Plachouri and Georgiou, emphasize tailoring therapy to lesion size, site, and patient preference [10].

In our patient, shave excision followed by electrocautery was selected, balancing effectiveness with cosmetic outcomes and the need for prompt resolution. Unlike most eyebrow PGs, which are reported in younger individuals [1,2], our patient was 50 years old. Additionally, her lesion caused significant functional impairment by obstructing vision. These features distinguish this case from the majority of previously published reports.

## Conclusions

Although PG is a frequent benign vascular lesion, eyebrow involvement is rarely encountered. This case underscores the importance of including PG in the differential diagnosis of unusual vascular nodules, particularly in cosmetically and functionally significant regions. Histological assessment remains essential for confirmation, and complete excision ensures both diagnostic certainty and excellent therapeutic results with minimal recurrence. Moreover, documenting such atypical presentations raises awareness among clinicians and highlights the need for careful examination of uncommon sites. It also emphasizes the value of individualized treatment approaches that consider both medical efficacy and cosmetic outcomes.
